# Assessment of Coagulation Parameters in Women Affected by Endometriosis: Validation Study and Systematic Review of the Literature

**DOI:** 10.3390/diagnostics10080567

**Published:** 2020-08-07

**Authors:** Jessica Ottolina, Ludovica Bartiromo, Carolina Dolci, Noemi Salmeri, Matteo Schimberni, Roberta Villanacci, Paola Viganò, Massimo Candiani

**Affiliations:** 1 Gynecology/Obstetrics Unit, IRCCS San Raffaele Scientific Institute, 20132 Milan, Italy; ottolina.jessica@hsr.it (J.O.); bartiromo.ludovica@hsr.it (L.B.); dolci.carolina@hsr.it (C.D.); salmeri.noemi@hsr.it (N.S.); schimberni.matteo@hsr.it (M.S.); villanacci.roberta@hsr.it (R.V.); candiani.massimo@hsr.it (M.C.); 2 Reproductive Sciences Lab, Division of Genetics and Cell Biology, IRCCS San Raffaele Scientific Institute, 20132 Milan, Italy

**Keywords:** endometriosis, coagulation, thrombin time, activated partial thromboplastin time, platelet-to-lymphocyte ratio, neutrophil-to-lymphocyte ratio

## Abstract

The presence of endometriosis determines an inflammatory response locally. The objective of this validation study and systematic review was to assess systemic levels of coagulation and inflammatory parameters in women with or without the disease. We conducted a retrospective analysis of a database prospectively collected from January 2017 to February 2020 including *n* = 572 women who underwent laparoscopic surgery for endometriosis (cases, *n* = 324) or other benign gynecologic diseases (controls, *n* = 248). Inflammatory markers and coagulation parameters were determined. An advanced systematic search of the literature on the same parameters was conducted up to April 2020. A significantly higher neutrophil count was found in endometriosis patients. Patients with endometriomas and stage III–IV disease had a significantly lower absolute lymphocyte count and shortened activated partial thromboplastin time (aPTT) values. In the final regression model, aPTT retained significant predictive value for stage III–IV endometriosis (odds ratio (OR) = 0.002, 95% confidence interval (CI) = 0.00–0.445; *p* = 0.024). Results from the *n* = 14 included studies in the systematic review are characterized by a high variability, but some consistency has been found for alterations in thrombin time, platelet-to-lymphocyte ratio, and neutrophil count associated with endometriosis. Modest systemic changes of some inflammatory and coagulation parameters are associated with endometriosis. Indeed, all the modifications detected are still within the normal reference intervals, explaining the high heterogeneity among studies.

## 1. Introduction

Endometriosis, defined as the presence of endometrial tissue and fibrosis located outside the uterine cavity, is a common chronic disease that affects around 10% of women of reproductive age and is associated with infertility and pelvic pain [1,2,3,4]. Traditionally defined as a hormonal disease with an increased local production of estrogens due to aberrant steroidogenesis, it is also characterized by features of a pelvic chronic inflammatory condition. The presence of ectopic tissue in the peritoneal cavity is associated with overproduction of pro-inflammatory and pro-fibrotic cytokines and chemokines (i.e., interleukin (IL)-1β, IL-6, tumor necrosis factor (TNF)-α, and transforming growth factor-β (TGF-β)) detected in endometriotic lesions, endometriotic cyst fluid, and peritoneal fluid [5,6,7,8]. Macrophages infiltrating the ectopic lesions express typical markers of alternative activation, favoring the growth of the lesions and promoting their angiogenesis. Some inflammatory parameters, such as the neutrophil-to-lymphocyte ratio (NLR), have also been found elevated in the peripheral blood in patients with some forms of the disease [9,10]. The close association of inflammatory conditions and coagulatory processes has been known for a long time [11]. Platelets are the first immunomodulatory cells at the site of injury and inflammation, providing a functional link between host response and coagulation. Monocytes and neutrophils contribute to coagulation by the expression of tissue factor [7,8], which is upregulated upon inflammation. Other cells of the circulation and vasculature are altered by inflammatory conditions toward a pro-thrombotic state, as well. Moreover, in their activated state, neutrophils are capable of expelling neutrophil extracellular traps, which exert antibacterial functions, but also induce a strong coagulatory response. In line with the presence of a cross-talk between these two systems, platelet count (PLC) has been found to be increased in patients affected by endometriosis [12,13], while activated partial thromboplastin time (aPTT) and thrombin time (TT) were shown to be shortened [12,14]. In 2018, our group specifically demonstrated that endometriosis patients had a significantly shorter aPTT than women not affected by the disease and, in the subgroup analysis, women with ovarian disease had significantly shortened aPTT values in comparison to women without this form. Furthermore, both platelet-to-lymphocyte ratio (PLR) and aPTT were shown to be altered in the less severe forms. Since endometriotic cells express tissue factor (TF), these alterations were suggested to represent the subtle manifestation of the activation of this factor in the lesions and were portrayed in the context of angiogenesis and, thus, the development and progression of the disease [15]. Based on this evidence, coagulation and inflammatory parameters have also been proposed as systemic biomarkers for the presence of endometriosis. However, although their values seem to be significantly different from controls, they still remain in the normal range. In light of these data, other evidence is needed in order to confirm the presence of subtle alterations of coagulation parameters in endometriosis before setting up investigations on the pathogenetic and clinical significance of these findings. We have herein analyzed systemic levels of coagulation and inflammatory parameters in a validation study including women with or without endometriosis undergoing gynecologic pelvic surgery. In addition, to offer a general view of available data, we systematically reviewed and compared our findings with results from the current literature focused on this topic.

## 2. Materials and Methods

### 2.1. Retrospective Case–Control Study

This study was based on a retrospective analysis of a surgical database prospectively collected from January 2017 to February 2020 at San Raffaele Scientific Institute in Milan, Italy. All patients had a surgical indication for gynecologic diseases and underwent laparoscopic surgery. All participants met the following inclusion criteria: non-pregnant, reproductive-age women; normal hepatic and renal function tests; and a surgical indication for endometriosis or other benign gynecologic diseases. Women with coagulation disorders, autoimmune diseases, diagnosis of uterine or ovarian malignancy, or concomitant use of antiplatelet or anticoagulant therapy at the time of surgery were excluded. Women whose data on coagulation status were not available were also excluded. Information about age, body mass index (BMI), smoking status, medical history, previous history of gynecological surgery, intraoperative findings, histopathological diagnosis, and routine blood tests were collected. The routine preoperative tests included complete blood count parameters, NLR, PLR, PT (prothrombin time) ratio, aPTT ratio, and international normalized ratio (INR). A peripheral blood sample (2 mL) was obtained from the median cubital vein of each patient and mixed with 3.2% citric acid for anticoagulation purposes. All blood analyses were obtained at a maximum of 1 month before surgery. The NLR was obtained by dividing the absolute neutrophil count by the absolute lymphocyte count, while the PLR was obtained by dividing the absolute platelet count by the absolute lymphocyte count. All blood analyses were done during either the follicular or the luteal phase of the cycle before surgery. The case group included patients with a diagnosis of endometriosis. The stage of endometriosis was established according to the revised American Fertility Society (r-AFS) classification [16]. Endometriotic lesions were classified according to their phenotype as ovarian endometrioma (OMA), deep infiltrating endometriosis (DIE), and superficial peritoneal endometriosis (SPE) [17]. Since these phenotypes are frequently combined, patients were assigned to the group corresponding to the most severe lesion detected, with the severity scale going from the least to the most severe as follows: SPE, OMA, DIE. The control group consisted of women with a surgical diagnosis of tubal pathology and ovarian benign cysts. Both the surgical and the histopathological examinations confirmed no evidence of endometriosis in the control population. According to the abovementioned selection criteria, *n* = 572 women were included: *n* = 324 had a diagnosis of endometriosis, and *n* = 248 had a diagnosis of other gynecologic diseases. All the women signed a written informed consent to record their data for scientific purposes. The Institutional Review Board of our Institution approved the study (Comitato Etico Ospedale San Raffaele; No. 01END, approved 12 April 2012).

### 2.2. Systematic Review of the Literature

The study was registered and accepted for inclusion in the database PROSPERO (ID CRD42020171524). The systematic review was carried out in accordance with the methods proposed by Preferred Reporting Item for Systematic Reviews and Meta-analysis (PRISMA) guidelines [18]. We performed an advanced, systematic search of online medical databases PubMed and Medline using the following keywords: “endometriosis” in combination with “thrombin”, “thrombin time”, “thromboplastin”, “partial thromboplastin time”, “activated partial thromboplastin time”, “INR”, “international normalized ratio”, “coagulation/blood coagulation”, “platelets/blood platelets”, “lymphocyte”, “platelets-to-lymphocyte ratio/platelets-lymphocyte ratio”, or “neutrophil-to-lymphocyte ratio/ neutrophil-lymphocyte ratio”. To optimize search output, we used specific tools available in each database, such as Medical Subject Headings (MeSH) terms (PubMed/Medline). The EndNote software (available online: https://endnote.com, accessed on 31 May 2020) was used to remove duplicate articles. Only full-length manuscripts written in English up to April 2020 were considered. We checked all citations found by title and abstract to establish the eligibility of the source and obtained the full text of eligible articles. We also performed a manual scan of the references list of the review articles to identify any additional relevant citations. Three review authors (J.O., M.S., and L.B.) independently assessed the risk of bias for each study using the risk-of-bias tool for case–control studies developed by clarity group [19]. We assessed the risk of bias according to the following domains: i) Can we be confident in the assessment of exposure?; ii) Can we be confident that cases had developed the outcome of interest and controls had not?; iii) Were the cases properly selected?; iv) Were the controls properly selected?; v) Were cases and controls matched according to important prognostic variables or was statistical adjustment carried out for those variables?. We graded each potential source of bias as Definitely yes (low risk of bias), Probably yes (Moderate risk of bias), Probably no (Serious risk of bias), or Definitely no (Critical, high risk of bias). We summarized the risk of bias judgments across different studies for each of the domains listed.

### 2.3. Statistical Analysis

Statistical analysis was performed using IBM SPSS Statistics, Version Chicago 24.0 (IBM Corp. Realesd 2016. Version 24.0. Armonk, NY, USA). Differences in systemic inflammatory response markers between cases and controls were investigated. Coagulation parameters were analyzed including only patients who were not taking any hormonal therapy at the time of surgery. Categorical variables were expressed as absolute value and percentages, and between-groups comparisons were evaluated using the Pearson’s chi square test with a Monte Carlo approximation at 95% confidence interval (CI). Continuous and normally distributed variables were presented as mean, range, and standard deviation (SD), and between-groups differences were investigated using the independent Student’s t-test. Subgroup analyses according to the stage and type of endometriosis were performed using the one-way analysis of variance (ANOVA) test. Before conducting means comparisons, the assumption of homogeneity of variances was tested and satisfied based on Levene’s F tests. In order to evaluate the nature of the differences between the means further, each statistically significant ANOVA test was followed-up with a Bonferroni’s post hoc test. A binary logistic regression was conducted in order to evaluate coagulation and inflammatory parameters as independent predictor factors of endometriosis. Adjusted odds ratios with 95% CI were evaluated when a statistically significant correlation was found. *p*-values < 0.05 were considered statistically significant.

## 3. Results

### 3.1. Results of the Retrospective Analysis

Of the *n* = 324 women affected by endometriosis, *n* = 85 were stage I or II (26.2%) disease, whereas the remaining *n* = 239 patients (73.76%) were stages III or IV. According to the type of disease, *n* = 214 patients (66%) were classified as having OMA, *n* = 69 patients (21.3%) as having DIE, and *n* = 41 patients (12.7%) as having SPE. Endometriosis could not be detected in *n* = 248 women. These cases were used as controls. The main diagnosis of this group was as follows: ovarian dermoids (*n* = 110), serous or seromucinous ovarian cysts (*n* = 77), tubal pathology (*n* = 43), and normal pelvis (*n* = 18). The baseline characteristics of patients with and without endometriosis are shown in Table 1.

In line with previous observations [20], patients with endometriosis had a significantly lower BMI compared with controls; moreover, patients with endometriosis were older than non-endometriosis patients. Results from comparisons of systemic inflammatory parameters between cases and controls are reported in Table 2. A significantly higher neutrophil count was found in patients with endometriosis when compared to controls. No difference in lymphocytes count and NLR was detected between the two groups. When we considered the various manifestations of endometriosis separately, we found that women with ovarian disease had a borderline significant lower absolute lymphocyte count in comparison with controls, SPE group, and DIE group. In addition, women with stage III to IV disease had a slightly lower lymphocyte count than those with stage I to II disease, and the difference reached statistical significance. In order to evaluate the real effect of endometriosis on the coagulation status, we decided to include in the comparisons only cases (*n* = 163) and controls (*n* = 96) who were not taking any hormonal therapy at the time of surgery because of the well-known impact of the treatment on the coagulation parameters [21,22]. Intergroups differences of coagulation parameters and inflammatory response markers in patients without hormonal therapy are shown in Table 3.

**Figure 1:**
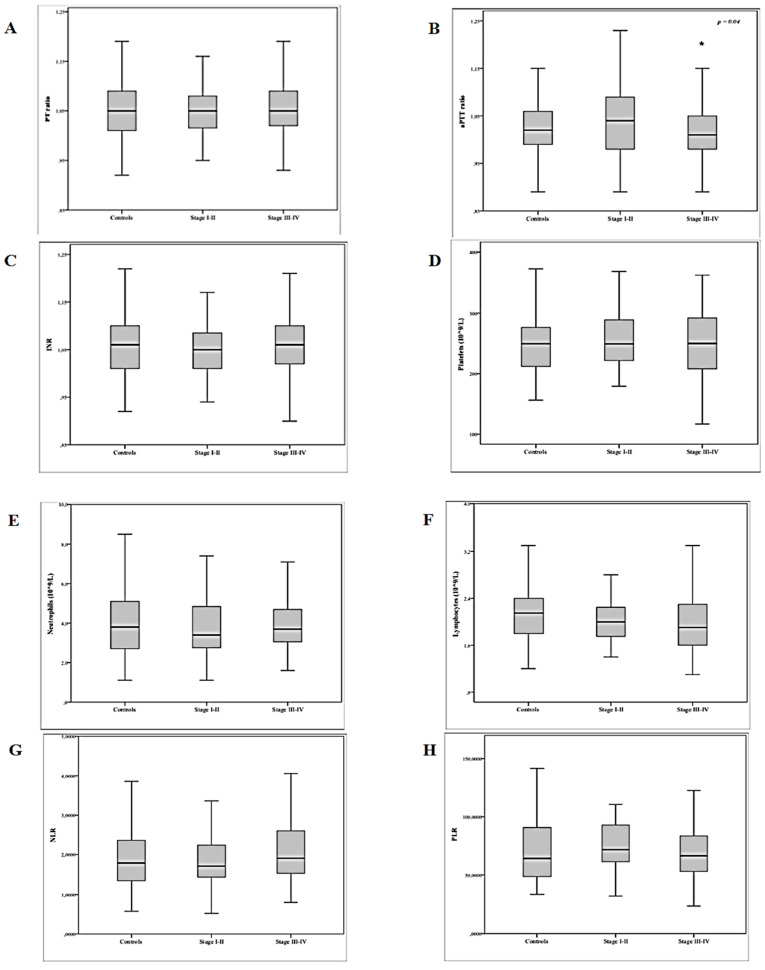
Boxplots of levels of coagulation parameters and systemic inflammatory response parameters according to different stages of endometriosis (*n* = 259). (**A**) PT, prothrombin time; (**B**) aPTT, activated partial thromboplastin time; (**C**) INR, international normalized ratio; (**D**) PLC, platelet count; (**E**) N, neutrophil count; (**F**) L, lymphocyte count; (**G**) NLR, neutrophil-to-lymphocyte ratio; (**H**) PLR, platelet-to-lymphocyte ratio. * *p* = 0.04.

For inflammatory parameters, in line with the above reported results, patients with OMAs had significantly lower absolute lymphocytes count if compared to both controls and DIE group. No difference in neutrophils count and NLR was detected among the endometriosis phenotypes. Focusing on the coagulation parameters, a significant between-group difference emerged in aPTT values, as women with OMA disease had shortened aPTT values if compared to patients with SPE, DIE, and controls. Moreover, women with stage III–IV disease had slightly, but significantly, shorter aPTT values than those with stage I–II endometriosis or than controls. No difference was found for platelet count or PLR among the various groups. Boxplots of levels of coagulation parameters and systemic inflammatory response markers according to the different stages of endometriosis are presented in Figure 1. A logistic regression was conducted in order to evaluate whether a certain coagulation or inflammatory status could be a predictor of the disease. The binary logistic regression was able to correctly classify 78.5% of cases (R^2^ = 0.05, χ^2^(1) = 5.29, *p* = 0.021). In the final regression model, aPTT retained significant predictive value for stages III–IV endometriosis (*b* = −6.091, standard error (SE) = 2.695; OR = 0.002, 95% CI = 0.00–0.445; *p* = 0.024) (Table 4).

### 3.2. Results of the Systematic Review

**Figure 2:**
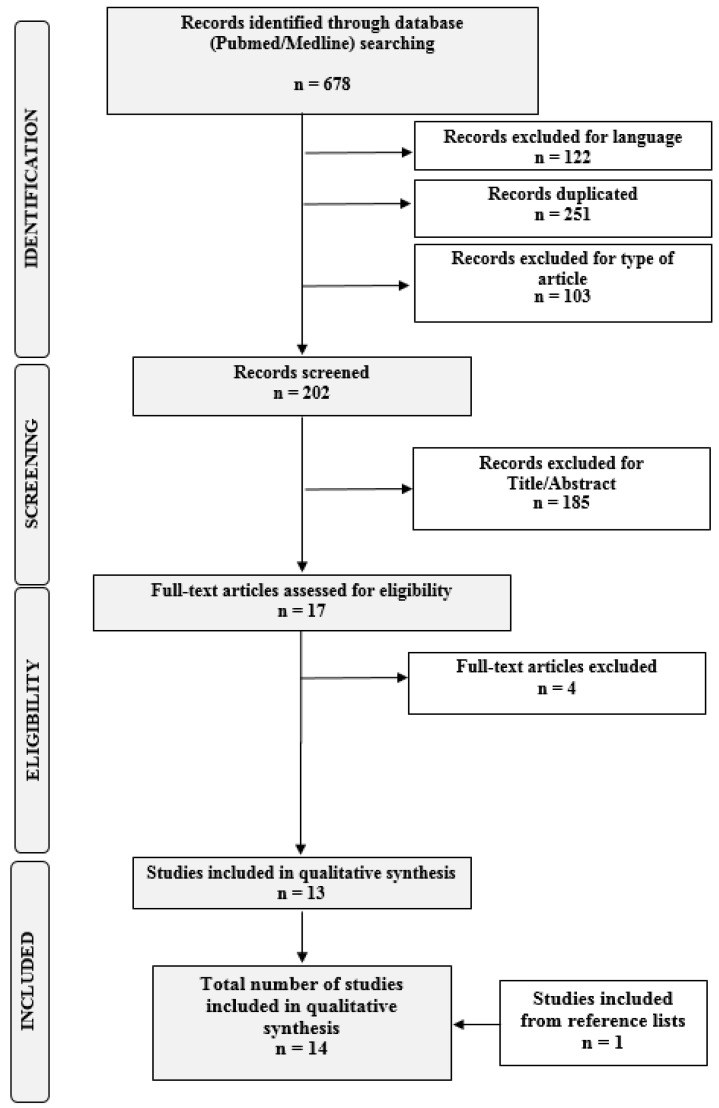
Flow diagram of the search strategy, screening, eligibility, and inclusion criteria.

The search revealed *n* = 17 studies eligible for inclusion in this systematic review. Of these, *n* = 14 were finally included [9,12,13,14,23,24,25,26,27,28,29,30,31,32]. A flow diagram of the systematic review is shown in Figure 2 (PRISMA template). The main characteristics of the included studies are summarized in Table 5. The risks of bias of the included studies are summarized in Supplementary Figure S1.

#### 3.2.1. Coagulation Parameters in Endometriosis Patients

Table 6 shows the results of the studies that have investigated coagulation parameters in relation to the presence of endometriosis. The first studies that evaluated PLC did not find significant differences between endometriosis patients and the control groups [24,25]. The same results were obtained in other studies addressing PLC as a secondary outcome in the investigations of other coagulation parameters useful for the diagnosis of endometriosis [9,12,13,14,31]. In a retrospective case–control study that included women with adenomyosis, endometriosis, and a control group, Coskun et al. found a statistically higher PLC in endometriosis patients versus controls [32]. Similar results were obtained by Seckin and coworkers that, considering women with OMAs or other benign adnexal cysts, reported a significantly higher PLC in the OMA group compared to controls, and this difference remained even when considering the younger (<25 years old) and older (>25 years old) subgroups [28]. Avcioğlu et al. found that in patients with advanced endometriosis (stages III–IV), PLC was significantly higher when compared to minimal–mild endometriosis (stages I–II) and showed a significant positive correlation between PLC (*r* = 0.8; *p* = 0.001) and white blood cell (WBC) [26]. This finding was later supported by Ding et al., who reported a significantly higher PLC in women with endometriosis compared to controls and, within the OMA group, a significantly higher PLC mean value in case of severe endometriosis [30]. Opposite results were obtained by Kim and coworkers, since PLC was not significantly different in severe endometriosis [27]. Three studies have evaluated PT [12,29,30], and only Ding and coworkers showed a significantly shorter time in the OMA group compared to a benign cyst group and a control group [30]. Conversely, for TT, three studies [12,29,30] out of four showed significantly shorter values in patients affected by OMAs compared to control groups [12,14,29,30]. Three studies have evaluated differences in INR between patients with and without endometriosis finding no difference [12,14,29]. Four studies have addressed aPTT [12,14,29,30], and two of them have reported shortened aPTT in cases when compared to controls [12,14]. In particular, in our previous study, considering the various manifestations of endometriosis separately, we found that women with ovarian disease had shortened aPTT values in comparison to controls and women with SPE and DIE. In addition, women with stage I to II endometriosis had slightly shorter, but significant, aPTT values than those with stage III to IV disease [14]. In a multivariate logistic regression analysis, after controlling for potential confounders (age, parity, BMI, and smoking), aPTT retained significant predictive value for endometriosis [14]. Interestingly, in a cross-sectional study considering 100 women with OMA before and three months after surgery, Ding and colleagues found that, after the surgical removal of all visible lesions by laparoscopy, the coagulation measurements (PLC, INR, PT, aPTT, and TT) were all significantly changed suggesting a possible role for active endometriotic lesions in this modification [29].

#### 3.2.2. Systemic Inflammatory Markers in Endometriosis Patients

Results for the inflammatory markers, including neutrophils, lymphocytes, PLR, and NLR, from the systematic review are presented in Table 7. Three studies showed that neutrophil count was higher in patients with endometriosis than in women without the disease [9,23,31]. On the other hand, three studies showed no significant difference among groups, either when considering OMAs or in non-OMA patients [14,25,28]. Importantly, our present study confirmed a significantly higher neutrophil count in patients with endometriosis. Among eight studies investigating the lymphocyte count [9,14,23,24,25,27,28,31], only three of them reported a significantly lower mean cell count in endometriosis compared to controls [9,23,31], and no correlation between the stage of endometriosis and the lymphocyte count was observed by Kim et al. [27]. Two studies found no difference between cases and controls in terms of NLR [25,28]. In our previous study, we found no difference in NLR between women with endometriosis and controls, although, when considering the various manifestations of endometriosis separately, a significant difference among groups emerged, as women with peritoneal lesions had lower NLR compared to patients without this form [14]. On the contrary, NLR was found to be significantly increased in the endometriosis group by Cho et al., who evaluated the usefulness of NLR in diagnosing endometriosis compared to benign ovarian tumors and healthy controls [23]. The same result was subsequently confirmed by three other studies [9,30,31]. Kim and coworkers, comparing stage I–II to stage III–IV endometriosis cases who underwent laparoscopic conservative surgery for OMAs, did not find any difference in NLR [27]. Among six studies investigating PLR [9,14,24,25,28,30], results obtained were more consistent. Four groups reported a significantly higher PLR in women with endometriosis [9,24,28,30]. In our previous case–control study [14], considering the various manifestations of endometriosis separately, we found that women with stage I to II endometriosis had significantly higher PLR than those with stage III to IV disease. A higher PLR in stage III–IV of endometriosis has been reported by Yang et al., compared to benign adnexal tumors and controls [24]. Only the study by Yavuzcan et al. reported no statistically significant difference in terms of PLR between endometriosis patients and controls and among the various endometriosis subgroups [25].

## 4. Discussion

We have herein confirmed our previous results documenting the present of subtle alterations of the peripheral coagulation system in patients with endometriosis. More specifically, in the validation study, the sub-analysis according to the various forms of the disease showed a shortened aPTT to be associated with the presence of moderate–severe endometriosis and with the presence of OMAs. This result is in line with previous evidence demonstrating a shortened aPTT in women with endometriosis, in particular with the ovarian form [12,14].

Results from the systematic reviews are characterized by a high variability, but a certain rate of consistency has been found also for alterations in TT and PLR in association with endometriosis.

In relation to the inflammatory parameters, we found a significantly higher neutrophil count in patients with endometriosis and a significantly lower absolute lymphocyte count in women affected by OMAs. We failed to detect similar results in our previous study thus supporting again the high variability of the observations. On the other hand, in line with our present findings, some groups had already reported that neutrophil count and NLR were significantly increased in the endometriosis group [9,23,30,31].

Variability of the results among the different studies may have different explanations. First, numbers of cases enrolled in the various studies are limited and, given the small changes observed among groups, the possibility of detecting significant differences is reduced. This is the reason for our choice to proceed with a systematic review. Second, the control groups are quite different among the studies, from only surgical patients as controls, or comparing women with other benign ovarian disease to endometriosis. A benign ovarian cyst may be responsible of an inflammatory pelvic environment as well, with a consequential alteration in inflammatory markers. Third, endometriosis is characterized by a plethora of manifestations and forms that are differently represented in the selected studies; some authors considered both minimal–mild and moderate–severe endometriosis, while other studies included only women with an advanced disease (stages III–IV) [27,30]. Similarly, some studies included only women without an ovarian disease while others considered only OMA patients [9,27,30] and others a combination of the two forms [25].

Overall, these results tend to confirm the idea that women with endometriosis are characterized by systemic changes of some inflammatory parameters [1,20,33,34] as well as by a modest change of the coagulation function. Indeed, all the modifications of the coagulation process detected are still within the normal reference intervals. Interestingly, Ding and coworkers have shown that three months after surgery for the removal of endometriotic lesions, the coagulation measurements were all significantly changed, suggesting a possible role for active endometriosis in the alterations of coagulation parameters, either locally or systemically [29].

The subtle variations observed in affected patients may be due to the TF pathway activation at the level of endometriotic lesions. Immunohistochemical studies revealed a marked elevation of TF expression pattern in eutopic and ectopic endometrium from women with endometriosis [35]. Moreover, the protease-activated receptor 2 (PAR-2), which is activated by TF/FVIIa, was as well demonstrated to be highly upregulated in the glandular epithelium of eutopic endometrium. Hence, both TF and the PAR-2 receptor are strategically poised for angiogenic and inflammatory signaling in endometriotic lesions [36,37]. Once TF is exposed to blood, it starts a reaction cascade that culminates in the increased production of thrombin. A shortened aPTT is correlated with elevated levels of coagulation factors (except factor VII) and of all markers for increased thrombin generation in plasma (prothrombin fragment 1,2, thrombin–antithrombin complexes, D-dimers, and factor VIII coagulant activity), thus determining a change in hemostatic balance in favor of a prothrombotic state [38,39]. In our multivariate logistic regression analysis, aPTT retained significant predictive value for stage III–IV endometriosis, but given the small difference detected, this diagnostic parameter is unlikely to be useful to fully differentiate women with and without disease. Endometriosis would not more frequently develop in women with shorter aPTT; however, we cannot exclude that these perturbations of the coagulation system may occur at some time during the pathogenetic process starting from repeated tissue repair lesions. Indeed, cyclic bleeding of endometriotic lesions determines the local release of factors such as those activating platelets (PAFs), thrombin and thromboxane A2 (TXA2), resulting in increased angiogenesis, increased vascular permeability and in the induction of platelet activation and aggregation [4,40,41,42]. The activated platelets would further release von Willebrand factor (vWF), adenosine diphosphate (ADP), serotonin, PAF, TXA2, and Chemokine (C-X-C motif) ligand 4 (CXCL4), causing further platelet aggregation and perpetuating the coagulation activation [43]. Importantly, the consequent extravasation and aggregation of platelets can finally induce fibrosis in endometriosis lesions through TGF-β1 release and induction of the TGF-β1/Smad3 signaling pathway, which is a potent inducer of epithelial–mesenchymal transition and fibroblast-to-myofibroblast transition in endometriotic cells [40,43,44].

Controversial data have been reported in relation to the platelet count among the various studies, while a quite consistent significantly higher PLR was observed in women in endometriosis. A possible explanation for this inconsistency is that platelets exert their role as “activated platelets”, without necessarily increasing their absolute mean value in peripheral blood. The elevated percentage of activated platelets in the peripheral blood of women with endometriosis is consistent with the observed shortened TT and aPTT. The release of TXA2, a potent platelet activation inducer, can generate a vicious cycle in maintaining platelet activation, the activation of the coagulation cascade, higher plasma fibrinogen levels, and short aPTTs in endometriosis [44,45].

Recently, increased cardiovascular and thrombotic morbidity in terms of myocardial infarction, angina, and coronary bypass graft intervention has been recognized in women with endometriosis. The relative risk of combined coronary heart disease events was 1.62 (95% confidence interval: 1.39–1.89) after adjustment for confounders [46,47]. Moreover, endometriosis has been interestingly identified as a novel predicting factor for venous thromboembolism during pregnancy and postpartum in a Japanese birth cohort study [48]. Factors contributing to this increased risk have not been deeply investigated. Possible causes could be the hormonal treatment or previous hysterectomy/oophorectomy in affected women. On the other hand, another hypothesis could attribute these events to these subtle alterations in coagulation and fibrinolysis parameters recently identified in affected patients causing a hypercoagulable status [12].

Our validation study has some limitations: (1) the retrospective design of the study that could have influenced the interpretation of the data; (2) patients on hormonal treatment have been included, but, in order to evaluate the unique effect of the endometriosis on the coagulation status, we did include only cases and controls with a negative history of hormonal therapy before surgery in the comparisons of coagulation parameters; (3) age was different between cases and controls; nevertheless, since a limited age range was set as a case selection criteria, a selection bias may be excluded.

## 5. Conclusions

In conclusion, our findings suggest that women with OMAs and moderate–severe forms of endometriosis show a modest strength of the coagulation function potentially attributable to the inflammatory nature of the lesions. Endometriosis also seems to be associated with systemic changes of some inflammatory parameters, for instance, a modest increase of neutrophil count. All the alterations detected are still within the normal reference intervals, explaining the high heterogeneity among studies. We cannot, however, rule out that these systemic perturbations may contribute to the pathogenetic process of the disease or to the increased cardiovascular and thrombotic morbidity observed in patients affected.

## Figures and Tables

**Table 1:** Baseline characteristics of the endometriosis and control groups.

Baseline Characteristics	Endometriosis Group (*n* = 324)	Control Group (*n* = 248)	*p*-Value
Age (years)	33.53 ± 5.51	31.15 ± 7.9	**0.001**
BMI (kg/m^2^)	21.37 ± 3.9	22.29 ± 4.0	**0.028**
Smoking habit	28 (14.7%)	35 (18.3%)	0.09
Indication for surgery			
Symptoms	174 (55.4%)	69 (29%)	
Offspring desire	113 (36%)	56 (23.5%)	**0.001**
Occasional findings	12 (3.8%)	104 (43.7%)	
Symptoms and offspring desire	15 (4.8%)	8 (3.4%)	
Prophylactic surgery		1 (0.4%)	
HT before surgery	128 (39.5%)	64 (25.8%)	0.43
Previous pelvic surgery	82 (25.9%)	85 (34.6%)	**0.027**

Note: values are mean ± SD or absolute value (%). Abbreviations: BMI, body mass index; HT, hormonal therapy. *p*-values < 0.05 were considered statistically significant (bold values).

**Table 2:** Systemic inflammatory response parameters according to the different stage and type of endometriosis versus controls.

Diagnosis	L (10^9^/L)	N (10^9^/L)	NLR
Endometriosis (*n* = 324)	2.05 ± 0.53	4.30 ± 1.51	2.21 ± 0.95
Controls (*n* = 248)	2.12 ± 0.55	4.03 ± 1.57	2.05 ± 1.50
	*p* = 0.13	*p* = **0.038**	*p* = 0.13
OMA (*n* = 214)	2.01 ± 0.52	4.32 ± 1.51	2.27 ± 0.97
DIE (*n* = 69)	2.19 ± 0.64	4.36 ± 1.51	2.12 ± 0.95
SPE (*n* = 41)	2.04 ± 0.56	4.08 ± 1.55	2.04 ± 0.78
Controls (*n* = 248)	2.12 ± 0.55	4.03 ± 1.57	2.05 ± 1.50
	*p* = **0.049**	*p* = 0.15	*p* = 0.27
Stage I–II (*n* = 85)	2.16 ± 0.56	4.29 ± 1.49	2.06 ± 0.81
Stage III–IV (*n* = 239)	2.01 ± 0.52	4.30 ± 1.52	2.25 ± 0.99
Controls (*n* = 248)	2.12 ± 0.55	4.03 ± 1.57	2.05 ± 1.50
	*p* = **0.032**	*p* = 0.11	*p* = 0.15

Note: values are mean ± SD or absolute value (%). Abbreviations: OMA, ovarian endometrioma; DIE, deep infiltrating endometriosis; SPE, superficial peritoneal endometriosis; N, neutrophil count; L, lymphocyte count; NLR, neutrophil-to-lymphocyte ratio. *p*-values < 0.05 were considered statistically significant (bold values).

**Table 3:** Coagulation parameters and systemic inflammatory response markers according to the different stage and type of endometriosis versus controls, excluding patients taking hormonal drugs.

Diagnosis	PT Ratio	aPTT Ratio	INR	PLC (10^9^/L)	L (10^9^/L)	N (10^9^/L)	NLR	PLR
OMA (*n* = 118)	1.05 ± 0.06	1.01 ± 0.07	1.06 ± 0.07	253.5 ± 62.0	1.93 ± 0.49	3.99 ± 1.48	2.18 ± 1.03	69.81 ± 25.6
DIE (*n* = 28)	1.05 ± 0.06	1.05 ± 0.08	1.05 ± 0.07	268.7 ± 73.0	2.29 ± 0.64	3.96 ± 1.27	1.79 ± 0.60	72.43 ± 22.7
SPE (*n* = 17)	1.07 ± 0.09	1.04 ± 0.07	1.07 ± 0.12	254.5 ± 53.1	1.97 ± 0.33	3.51 ± 1.26	1.80 ± 0.63	81.43 ± 33.0
Controls (*n* = 96)	1.05 ± 0.06	1.03 ± 0.06	1.06 ± 0.06	249.4 ± 50.2	2.11 ± 0.49	3.99 ± 1.57	1.99 ± 0.96	72.93 ± 37.6
	*p* = 0.61	*p* = **0.049**	*p* = 0.59	*p* = 0.50	*p* = **0.003** ^b^	*p* = 0.64	*p* = 0.117	*p* = 0.52
Stage I–II (*n* = 35)	1.06 ± 0.08	1.05 ± 0.08	1.06 ± 0.09	262.3 ± 64.7	2.12 ± 0.59	3.77 ± 1.36	1.83 ± 0.62	76.55 ± 27.4
Stage III–IV *(n =* 128)	1.02 ± 0.07	1.01 ± 0.07	1.06 ± 0.06	254.6 ± 62.8	1.97 ± 0.49	3.98 ± 1.45	2.14 ± 1.01	70.09 ± 25.6
Controls (*n* = 96)	1.05 ± 0.06	1.03 ± 0.06	1.06 ± 0.06	249.4 ± 50.2	2.11 ± 0.49	3.99 ± 1.57	1.99 ± 0.96	72.93 ± 37.6
	*p* = 0.78	*p* = **0.040** ^b^	*p* = 0.82	*p* = 0.53	*p* = 0.07	*p* = 0.73	*p* = 0.18	*p* = 0.51

Note: values are mean ± SD or absolute value (%). Abbreviations: OMA, ovarian endometrioma; DIE, deep infiltrating endometriosis; SPE, superficial peritoneal endometriosis; PT, prothrombin time; aPTT, activated partial thromboplastin time; INR, international normalized ratio; PLC, platelet count; N, neutrophil count; L, lymphocyte count; NLR, neutrophil-to-lymphocyte ratio; PLR, platelet-to-lymphocyte ratio. ^b^ When *p* < 0.05, a Bonferroni’s post hoc test was performed for within-groups differences. *p*-values < 0.05 were considered statistically significant (bold values).

**Table 4:** Logistic regression of coagulation and inflammatory parameters predicting the stage of endometriosis.

Variable	B Coefficient	SE	OR	*p*-Value
PT ratio	−4.084	12.301		0.74
aPTT ratio	−6.091	2.695	**0.002**	**0.024**
INR	3.611	11.279		0.75
PLC (10^9^/L)	0.000	0.006		0.95
L (10^9^/L)	−0.587	0.728		0.42
N (10^9^/L)	0.645	1.355		0.63
NLR	1.342	1.402		0.34
PLR	−0.007	0.018		0.71

Abbreviations: SE, standard error; OR, odds ratio; PT, prothrombin time; aPTT, activated partial thromboplastin time; INR, international normalized ratio; PLC, platelet count; N, neutrophil count; L, lymphocyte count; NLR, neutrophil-to-lymphocyte ratio; PLR, platelet-to-lymphocyte ratio. *p*-values < 0.05 were considered statistically significant (bold values).

**Table 5:** Main characteristics of considered studies.

Author, Years	Country	Study Design	Study Period	Cases/Controls Sample Size (*n*)	Age (Years)	Parameters Assessed (When)	Confounding Factors
Cho et al., 2008 [23]	South Korea	Retrospective case–control study	01/2004 12/2007	Endometriosis ^1^ (231)/ Benign ovarian cysts ^1^ (231) Healthy women ^2^ (384)	33.3 ± 7.3 * (Overall) 32.6 ± 7.35 * (endometriosis) 34.2 ± 8.9 * (benign ovarian cyst) 33.9 ± 5.7 * (healthy women)	Complete blood cell count, NLR, and CA125 (before surgery or as part of routine health examination ^3^)	Unclear
Yavuzcan et al., 2013 [25]	Turkey	Retrospective case–control study	11/2009 02/2013	Endometriosis ^1^ stage III/IV (61) - 33 with OMA - 28 non-OMA/Tubal ligation ^1^ (33)	36.21 ± 8.37 * (Overall)	Complete blood cell count, NLR, PLR, and CA125 (before surgery ^3^)	No
Avcioğlu et al., 2014 [26]	Turkey	Retrospective study	01/2001 06/2013	Endometriosi ^1^ stage III/IV (124)/Endometriosis ^1^ stage I/II (40)	33.7 ± 7.7 * (Overall)	Complete blood cell count, MPV, PDW, and PCT (before surgery ^3^)	No
Kim et al., 2014 [27]	South Korea	Retrospective study	04/2005 03/2013	Endometriosis ^1^ stage III (189)/Endometriosis ^1^ stage IV (230)	15–51 (Overall) 19–49 - 33.8 ± 6.8 * (stage III) 15–51 - 34.7 ± 7.0 * (stage IV)	Complete blood cell count, NLR, CRP, AMH, CEA, CA125, CA 19-9 (<1 month before surgery)	No
Chmaj-Wierzchowska et al., 2015 [13]	Poland	Hospital-based case–control	09/2009 11/2012	OMA ^1^ without coexisting foci of peritoneal endometriosis (48)/Mature teratomas ^1^ (38)	18–38 (Overall) 30.00 ± 4.6 * (OMA) 27.03 ± 4.52 * (teratomas)	Complete blood cell count, fibrinogen, urocortin, ghrelin, and leptin (<1 day before surgery)	No
Yang et al., 2015 [24]	China	Retrospective case–control study	01/2009 06/2012 ^4^	Endometriosis ^1^ - 119 Stage III - 78 Stage IV/ Benign ovarian cysts ^1^ (102) Healthy women ^2^ (112)	32.58 ± 6.37 * (Overall) 32.17 ± 6.50 * (endometriosis) 32.03 ± 6.83 * (benign ovarian cyst) 33.81 ± 5.52 * (healthy women)	Complete blood cell count, PLR, and CA125 (before surgery or as part of routine health examination ^3^)	Unclear
Wu et al., 2015 [12]	China	Hospital-based case–control	06-12/2012	OMA ^1^ (50) -35 stage III −15 stage IV/Age-matched healthy women ^2^ (50)	23–44 – 32.9 ± 6.1 * (OMA) 20–48 – 31.4 ± 6.4 * (controls)	Complete blood cell count, aPTT, PT, TT, INR, fibrinogen, D-dimer, fasting serum glucose, and serum cortisol (before surgery ^3^)	Yes
Tokmak et al., 2016 [9]	Turkey	Retrospective case–control study	01/2008 01/2013	OMA ^1^(467)/Age- and BMI-matched benign ovarian cysts ^1^ (340)	16–50 (Overall) 18–49 – 33.7 ± 8.4 * (OMA) 16–50 - 33.9 ± 11.6 * (Controls)	Complete blood cell count, NLR, PLR, CA125, AFP, CA 19-9, CA-15.3 (<1 month before surgery)	Unclear
Ding et al., 2018 [14]	China	Cross-sectional study	04/2015 03/2016	OMA ^1^(100)/ Women without endometriosis (100): - 60 Healthy women ^2^ - 40 CINIII or ovarian teratoma ^1^	21–49 (Overall) 32.0 ± 7.1 * (OMA) 33.0 ± 7.1 * (controls)	PLC, platelet activation rate, maximum platelet aggregation rate, D-dimer, fibrinogen, FDPs, sP-sel, F1 + 2, PT; TT; aPTT, INR (before surgery ^3^ and 3 months later only in OMA)	Unclear
Seckin et al., 2018 [28]	Turkey	Retrospective case–control study	01/2013 12/2016	OMA ^1^ (267)/ Benign ovarian cysts ^1^ (235)	15–49 – 27.1 ± 7.2 * (overall) 28.3 ± 6.6 * (cases) 25.8 ± 7.6 * (controls)	Complete blood cell count, NLR, PCT, PDW, PLR, and CA125 (before surgery ^3^)	Yes (only age </> 25 years)
Viganò et al., 2018 [14]	Italy	Retrospective case–control study	01/2013 12/2015	Endometriosis ^1^ (169) - 45 Stage I–II - 124 Stage III–IV/ Benign gynecologic pathology ^1^ (145)	35.8 ± 5.9 * (endometriosis) 36.9 ± 6.5 * (controls)	Complete blood cell count, NLR, PLR, TT ratio, aPTT, and INR (<1 month before surgery)	Yes
Coskun et al., 2019 [32]	Turkey	Retrospective case–control study	01/2013 01/2015	Adenomyosis ^1^ (84) Endometriosis ^1^ (102)/ Tubal ligation ^1^ (88)	52.9 ±7.4 * (adenomyosis) 35.3 ± 8.7 * (OMA) 37.9 ± 4.2 * (Controls)	Complete blood cell count, MPV, and CA125 (<1 week before the surgery)	Unclear
Ding et al., 2019 [30]	China	Retrospective case–control study	06/2015 06/2017	OMA ^1^ (226)/ Cyst group ^1^ (210) Tubal reanastomosis ^1^ (112)	35.7 ± 0.4 * (OMA) 35.9 ± 0.4 * (Cyst group) 35.8 ± 0.5 * (Controls)	Complete blood cell count, TT, PT, fibrinogen, CRP, PLR, NLR, aPTT, and CA125 (<1 month before surgery)	Yes
Turgut et al., 2019 [31]	Turkey	Retrospective case–control study	01/2012 02/2017	Endometriosis ^1^ (121) - 17 Stage I–II - 104 Stage III–IV/ Healthy women ^2^ (136)	22–53 (endometriosis) 17–51 (controls)	Complete blood cell count, MPV, and CA125 (before surgery ^3^)	Yes (age)

*Note:* * Mean ± SD. ^1^ Women with surgical and pathological diagnosis of endometriosis or other benign diseases/conditions with exclusion of endometriosis; ^2^ no surgery performed; ^3^ not specified when; ^4^ healthy women recruited only between 1/2012 and 06/2012; OMA, ovarian endometriosis; NLR, neutrophil–lymphocyte ratio; CA125, cancer antigen 125; PLR, platelet–lymphocyte ratio; MPV, mean platelet volume; PDW, platelet distribution width; PCT, plateletcrit; CPR, C-reactive protein; AMH, anti-Müllerian hormone; CEA, carcinoembryonic antigen; CA19-9, cancer antigen 19-9; aPTT, activated partial thromboplastin time; PT, prothrombin time; TT, thrombin time; INR, international normalized ratio; AFP, a- fetoprotein; CA15-3, cancer antigen 15-3; PLC, platelets; FDPs, fibrin degradation products; sP-sel, plasma-soluble P-selectin; F1 + 2, prothrombin fragment F1 + 2.

**Table 6:** Systematic review: coagulation parameters of women with and without endometriosis in the included studies.

Author	Year	Study Population (*n*)	PT	TT	aPTT	PLC (10^9^/L)	INR
Yavuzcan et al. [25]	2013	Cases (OMA): 33				269.8 ± 65.2	
		Cases (non-OMA): 28				298.9 ± 107.8	
		Controls: 33				286.4 ± 67.6	
Avcioğlu et al. [26]	2014	Stage I–II: 40				187 ± 36.18 *	
		Stage III–IV: 124				309.15 ± 54.43 *	
Kim et al. [27]	2014	Stage III (OMA): 189				NR	
		Stage IV (OMA): 230				NR	
Wu et al. [12]	2015	Cases (OMA): 50	NR	NR *	NR *	NR	NR
		Controls: 50	NR	NR *	NR *	NR	NR
Chmaj-Wierzchowska et al. [13]	2015	Cases (OMA): 48				267.80	
		Controls: 38				258.90	
Yang et al. [24]	2015	Cases: 197				253.25 ± 59.98	
		Benign tumor: 102				248.83 ± 61.69	
		Controls: 112				246.47 ± 52.55	
Tokmak et al. [9]	2016	Cases (OMA): 467				275.9 ± 72.1	
		Controls: 340				276.2 ± 71.3	
Seckin et al. [28]	2018	Cases (OMA): 267				292.9 ± 67.6 *	
		Controls: 235				269.7 ± 61.3 *	
Viganò et al. [14]	2018	Cases: 169		1.00 ± 0.9	1.12 ± 0.19 *	250.00 ± 55.8	0.99 ± 056
		Controls: 145		0.970 ± 0.10	1.13 ± 0.15 *	262.20 ± 63.4	0.98 ± 0.16
		Cases (OMA): 98		0.99 ± 0.06	1.08 ± 0.07 *	254.5 ± 61.47	0.99 ± 0.71
		Controls: 145		0.99 ± 0.09	1.14 ± 0.07 *	254.7 ± 58.62	0.99 ± 0.11
Ding et al. [29]	2018	Cases (OMA): 100	NR	NR *	NR	NR	NR
		Controls: 100	NR	NR *	NR	NR	NR
		Post-surgery (OMA): 100	NR *	NR *	NR *	NR *	NR *
Turgut et al. [31]	2019	Cases: 121				265 ± 86	
		Controls: 136				258 ± 70.5	
Ding et al. [30]	2019	Cases (OMA): 226	12.69 ± 0.04 *	15.42 ± 0.04 *	NR	239.8 ± 3.6 *	
		Controls: 112	12.99 ± 0.06 *	15.78 ± 0.06 *	NR	220.0 ± 5.4 *	
		Benign cyst: 210	13.00 ± 0.04 *	15.68 ± 0.05 *	NR	228.4 ± 4.0 *	
		Stage III (OMA): 91	12.64 ± 0.06	15.38 ± 0.06	35.68 ± 0.30	243.8 ± 5.4 *	
		Stage IV (OMA): 135	12.72 ± 0.05	15.44 ± 0.05	35.44 ± 0.26	237.1 ± 4.7 *	
		Coskun et al. [32]	2019	Cases: 102				292.9 ± 73.9 *	
Adenomyosis: 84						295.1 ± 77.5	
Controls: 88						269.9 ± 59 *	

*Note:* * statistically significant; PT: prothrombin time; TT: thrombin time; aPTT: activated partial thromboplastin time; PLC: platelet count; INR: international normalized ratio; OMA: ovarian endometrioma; NR: not reported.

**Table 7:** Systematic review: inflammatory parameters of women with or without endometriosis in the included studies.

Author	Year	Study Population (*n*)	Neutrophils 10^9^/L	Lymphocytes 10^9^/L	NLR	PLR
Cho et al. [23]	2008	Cases: 231	4.41 *	1.82 *	2.66 *	
		Benign tumor: 145	4.17 *	1.96 *	2.31 *	
		Controls: 384	3.6 *	1.95 *	1.99 *	
Yavuzcan et al. [25]	2013	Cases (OMA): 33	4.14 ± 1.73	2.12 ± 0.87	2.40 ± 2.04	162.84 ± 141.28
		Cases (non-OMA): 28	4.68 ± 2.18	2.02 ± 0.68	2.51 ± 1.37	159.14 ± 61.20
		Controls:33	4.50 ± 1.57	2.25 ± 0.66	2.11 ± 0.86	132.45 ± 35.74
Kim et al. [27]	*2014*	Stage III (OMA): 189		NR	NR	
		Stage IV (OMA):230		NR	NR	
Yang et al. [24]	*2015*	Cases: 197		1.91 ± 0.52		141.79 ± 51.78 *
		Benign tumor:102		2.02 ± 0.52		129.28 ± 39.20 *
		Controls: 112		2.05 ± 0.49		126.68 ± 39.67 *
Tokmak et al. [9]	*2016*	Cases (OMA): 467	4.8 ± 1.8 *	1.98 ± 5.92 *	2.8 ± 2.0 *	153.3 ± 71.3 *
		Controls: 340	3.8 ± 1.2 *	2.41 ± 7.17 *	1.7 ± 0.5 *	122.4 ± 42.7 *
Viganò et al. [14]	*2018*	Cases: 169	3.76 ± 1.34	2.04 ± 0.56	NR	NR
		Cases (OMA): 98	3.9 ± 1.62	2.02 ± 0.66	2.08 ± 1.01	135.18 ± 68.69
		Controls:145	3.99 ± 1.6	1.97 ± 0.55	2.16 ± 1.25	130.65 ± 52.8
Seckin et al. [28]	*2018*	Cases: 267	4.6 ± 1.8	2.2 ± 0.6	2.3 ± 1.3	142.3 ± 48.4 *
		Controls:235	4.5 ± 2.1	2.2 ± 0.7	2.1 ± 1.2	129.3 ± 40.4 *
Turgut et al. [31]	*2019*	Cases: 121	4.4 ± 1.9 *	2 ± 0.8 *	2.18 ± 0.86 *	
		Controls:136	3.55 ± 1.53 *	2.15 ± 0.8 *	1.70 ± 0.8 *	
Ding et al. [30]	*2019*	Cases: 226 (OMA)			2.56 ± 0.07 *	146.4 ± 2.8 *
		Benign cyst: 210			2.34 ± 0.07 *	137.7 ± 3.4 *

*Note:* * *p*-value statistically significant; OMA: ovarian endometrioma; NLR: neutrophil-to-lymphocyte ratio; PLR: platelet-to-lymphocyte ratio; NR: not reported.

## Supplementary Materials

The following are available online at https://www.mdpi.com/2075-4418/10/8/567/s1, Figure S1: Risk-of-bias assessment.
