# Supplementary material for: Assessment of Coagulation Parameters in Women Affected by Endometriosis: Validation Study and Systematic Review of the Literature
Source: Diagnostics (Basel). 2020 Aug 7;10(8):567. doi: 10.3390/diagnostics10080567 (PMC7460223; doi:10.3390/diagnostics10080567)
Supplement: Supplementary file 1 [file diagnostics-10-00567-s001.pdf]

Supplemental figure 1: Risk of bias assessment.

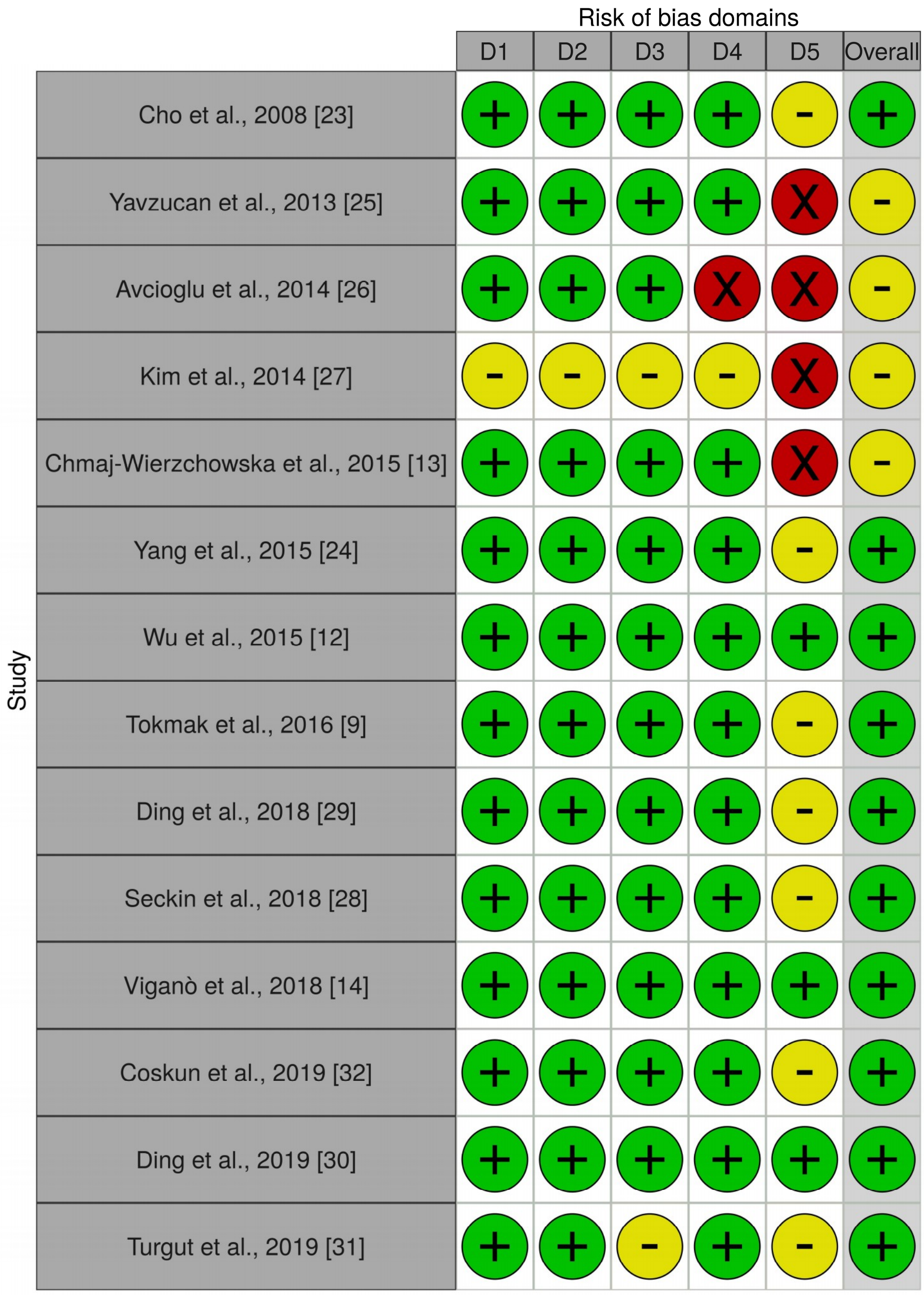

Notes: Green circle=low risk; yellow circle=unclear; red circle=critical; D1=Can we be confident in the assessment of exposure?; D2=Can we be confident that cases had developed the outcome of interest and controls had not?; D3=Were the cases properly selected?; D4=Were the controls properly selected?; D5=Were cases and controls matched according to important prognostic variables or was statistical adjustment carried out for those variables?.
